# Implementation of conditional reflex urine culturing decreases unnecessary antimicrobial use

**DOI:** 10.1017/ice.2025.10309

**Published:** 2025-12

**Authors:** Aoi Yogo, Elie A. Saade, Eric M. Ransom, Brigid M. Wilson, Timothy C. Jenkins, Abhishek Deshpande, Curtis J. Donskey, Zainab Albar, Lauren H. Epstein, Leila S. Hojat

**Affiliations:** 1 Department of Medicine, Case Western Reserve University School of Medicine, Cleveland, OH, USA; 2 Division of Infectious Diseases and HIV Medicine, University Hospitals Cleveland Medical Center, Cleveland, OH, USA; 3 Department of Pathology, Case Western Reserve University School of Medicine, Cleveland, OH, USA; 4 Department of Pathology, University Hospitals Cleveland Medical Center, Cleveland, OH, USA; 5 Geriatric Research, Education and Clinical Center, Louis Stokes Cleveland VA Medical Center, Cleveland, OH, USA; 6 Division of Infectious Diseases, Department of Medicine, Denver Health and Hospital Authority, Denver, CO, USA; 7 Division of Infectious Diseases, Department of Medicine, University of Colorado School of Medicine, Aurora, CO, USA; 8 Center for Value-Based Care Research, Cleveland Clinic, Cleveland, OH, USA; 9 Department of Infectious Diseases, Respiratory Institute, Cleveland Clinic, Cleveland, OH, USA; 10 Department of Medicine, Atlanta Veterans Affairs Health Care System, Decatur, GA, USA; 11 Division of Infectious Diseases, Emory University School of Medicine, Atlanta, GA, USA

## Abstract

**Objective:**

To evaluate the impact of implementation of a conditional reflex urine culturing strategy on urine culture rates, antimicrobial use, and clinical outcomes in hospitalized adults.

**Design:**

Pre-post quasi-experimental study.

**Setting:**

Emergency departments and inpatient units within a large, integrated healthcare system in Northeast Ohio, comprising 10 medical centers.

**Patients:**

Adult patients with a urine culture ordered from June 1, 2018, to May 31, 2023.

**Methods:**

A system-wide intervention was implemented on June 1, 2020, requiring urinalysis (UA) with pyuria findings to trigger a urine culture order. We compared urine culture rates, antimicrobial use (measured by days of therapy [DOT] and days of antimicrobial spectrum coverage [DASC]), and clinical outcomes between pre-and post-intervention periods.

**Results:**

The intervention resulted in an 85.4% reduction in urine culture rates (0.54 vs 3.71 per 100 patient days). Antimicrobial use decreased, with DOT per 100 patient days dropping by 11.5% and DASC/DOT by 16.1%. No significant differences were observed in *Clostridioides difficile* infection rate, subsequent bloodstream infections with urinary pathogens, or mortality between pre- and post-intervention groups.

**Conclusions:**

A conditional reflex urine culturing strategy implemented as part of a diagnostic stewardship framework reduced urine culture and antimicrobial use without adverse clinical outcomes. This highlights the potential of diagnostic stewardship to optimize antimicrobial use in hospitalized adults.

## Background

Urinary tract infection (UTI) is often misdiagnosed in patients presenting with asymptomatic bacteriuria (ASB), defined as the presence of bacteria in the urine without clinical manifestations of infection. The Infectious Diseases Society of America and the US Preventive Services Task Force recommend against testing for or treating ASB in general adult populations.^1,2^ Despite this guidance, urinalysis (UA) is frequently performed for patients without clinical signs or symptoms of UTI, increasing the probability of inappropriate urine culture and antimicrobial prescription.^3^

Diagnostic stewardship refers to procedures that modify test ordering, processing, or reporting to optimize diagnosis and downstream treatment, assisting providers with clinical diagnosis and treatment optimization.^4^ There are many potential applications for diagnostic stewardship in infectious diseases, such as conditional reflex urine culturing, also referred to as UA with reflex to urine culture, which is an automated laboratory-triggered testing strategy limiting urine culture performance to specimens meeting pre-specified abnormal UA parameters.^5,6^ Utilizing the presence of pyuria or other abnormal UA components as a prerequisite for urine culture has a high negative predictive value (NPV) for bacterial infection and has been associated with decreased urine culture rate.^7,8^ However, few studies have explored their effect on optimizing antimicrobial use and clinical outcomes.^8–13^ In this pre-post quasi-experimental study, we aim to comprehensively assess the impact of implementing a conditional reflex urine culturing strategy on urine culture rates, antimicrobial use, as well as relevant clinical outcomes in a large healthcare system.

## Methods

### Study design

We conducted a two-part study in a large, integrated healthcare system in Northeast Ohio, which included 10 medical centers after excluding hospitals that were not part of the system for the entire study period. The first aspect of the study involved a pre-post quasi-experimental design to compare urine culture and antimicrobial utilization pre- and post-intervention and included all adult patients (>18 years old) seen in the emergency department (ED) or hospitalized with a urine culture order from June 1, 2018, to May 31, 2023. Data from each patient including urine test orders, demographics, comorbidities, antimicrobial administration data, and *Clostridioides difficile* infection (CDI) testing were extracted from the electronic data warehouse (EDW), which is a repository of electronic medical record data, and were matched with urine culture data extracted from our microbiology database. Only patients with matching records in both systems were included in the study, and only the first culture in the first encounter was included for patients with multiple cultures in one or more encounters during the study period. The second part of the study included a retrospective evaluation of outcomes during the post-intervention period to determine potential negative impact of missed UTI among patients with negative UA and attempted urine culture by comparing these to patients with negative urine culture, using blood culture data obtained from our microbiology database and clinical outcomes data obtained from the EDW. This study was approved by the University Hospitals Institutional Review Board which waived the requirement for patient consent (STUDY20210303: Diagnostic stewardship).

### Intervention

We developed specific conditional urine culture indications: (1) pyuria with at least 5 white blood cells per high power field (WBCs/HPF) and (2) presence of nitrites, and/or presence of leukocyte esterase. We then applied these criteria to a system-wide intervention initiated on June 1, 2020.^7^ The intervention go-live date defined our two study phases: a pre-intervention period referring to all testing within the two years prior to this date and a post-intervention period referring to all testing within the three years after this date. During the post-intervention period, the prior stand-alone urine culture order was replaced with a new order for UA with reflex to urine culture. The order also required healthcare personnel to select a clinical indication for urine testing, including dysuria, urgency, frequency, fever, suprapubic pain, or flank pain. In addition to the conditional reflex urine culture order, a separate order called “urine culture for special populations” provided an option for urine testing in high-risk asymptomatic patients, including pre-urologic procedure, transplant, or pregnancy.^1,14^

### Urine culture rate and antimicrobial use assessment

**Figure 1.**
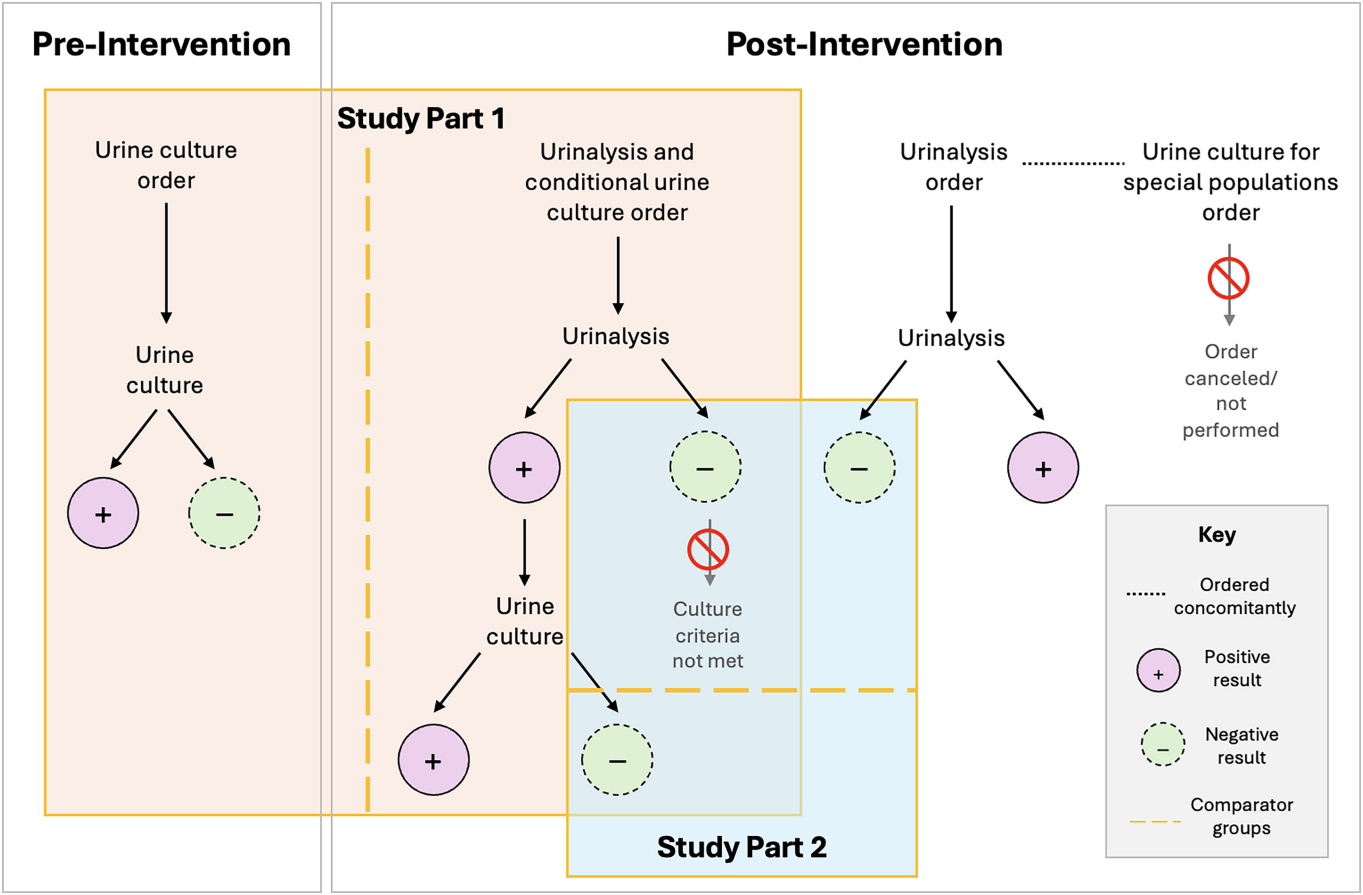
Diagram of approach to study design. Study Part 1 focuses on urine culture rates and antimicrobial use, while Part 2 evaluates potential negative clinical outcomes.

We compared urine culture rates and antimicrobial use among patients with urine culture performed during the pre-intervention period and UA with conditional culture order placed in the post-intervention period (Figure 1). Urine culture rate was defined as the number of urine cultures per total patient-days for all hospitalized patients during the study period. To describe antimicrobial use among patients who underwent urine testing, we utilized days of therapy (DOT) per 100 patient-days as well as the days of antimicrobial spectrum coverage (DASC) per DOT.^15^ We included the DASC metric to determine if patients who received antimicrobials were potentially receiving broad-spectrum therapy based on high clinical suspicion for infection without culture information. DASC as defined by Kakiuchi et al. were aggregated by daily antimicrobial spectrum coverage scores, which were calculated by 77 antimicrobial agents and organisms, including multidrug-resistant organisms (MDRO).^16^ Only antimicrobial use within the first 7 days after urine testing was evaluated to focus on treatment that may have been given or withheld directly as a result of the urine testing results. We also compared the rate of CDI pre- and post-intervention, hypothesizing that the intervention would be associated with reduced antimicrobial use, which in turn would lead to fewer cases of CDI.

### Negative outcomes assessment

To assess the potential negative impact of the intervention secondary to missed UTI diagnoses, we compared outcomes among patients in the post-intervention period including subsequent positive blood cultures with a typical urinary pathogen (defined as *Escherichia coli* or any species of the genera *Klebsiella*, *Enterobacter*, *Proteus*, *Enterococcus*, or *Pseudomonas*), total hospital length of stay, and mortality within 30 days of urine testing. For this analysis, we included patients in the post-intervention cohort with negative cultures (ie individuals with positive UA reflexed to urine culture later resulting as negative) and compared these to patients during the same period with a negative UA on the same date as an order for urine culture without a corresponding urine culture actually performed (ie UA orders which did not meet culture reflex criteria or a negative UA ordered along with a stand-alone urine culture for special populations with culture never performed) (Figure 1). This comparison was performed based on the premise that a patient with negative UA and no urine culture should be clinically comparable to a patient with a positive UA and negative urine culture.

### Analysis

Descriptive statistics were used to analyze the characteristics of each group. Urine culture rates by facility and by month were visualized. Rate ratios were determined for urine culture, DOT, and DASC in each study period by performing an exact test of the Poisson rates with confidence intervals obtained using the Clopper-Pearson method for binominal confidence intervals. Outcomes were compared using Wilcoxon rank sum test for continuous variables and Chi-square test for categorical variables. Odds ratios were calculated using median-unbiased estimation with confidence intervals calculated using mid-p exact test. Analyses were performed in R version 4.3.2. using the tidyverse (version 2.0.0) and epitools (version 0.5–10.1) packages.

## Results

We identified a total of 24,892 urine cultures during the study period, including 19,503 pre-intervention cultures and 5,389 post-intervention cultures (Table 1). Approximately 22% (n = 5,531) of urine cultures overall were positive. Patients were predominantly white, non-Hispanic females between 60 and 80 years of age, with few comorbidities. Notable differences in the post-intervention group included a higher proportion of females (60 vs 56%) and lower proportion of patients with immuno-compromised status (11 vs 18%).

**Table 1.** Characteristics of patients for whom urine culture was performed before and after the introduction of a conditional reflex urine culturing intervention. Values indicate total number and percentage unless otherwise noted (Study Part 1)

	Pre-intervention	Post-intervention	Overall
n	19,503	5,389	24,892
Male sex	8,625 (44.2)	2,147 (39.8)	10,772 (43.3)
Age (median [IQR])	70.0 [57.0, 81.0]	70.0 [56.0, 80.0]	70.0 [57.0, 81.0]
Race			
White	14,132 (72.5)	4,132 (76.7)	18,264 (73.4)
Black	3,946 (20.2)	1,047 (19.4)	4,993 (20.1)
Multiracial/other	187 (1.0)	55 (1.0)	242 (1.0)
AAPI	85 (0.4)	27 (0.5)	112 (0.4)
Indigenous American	18 (0.1)	5 (0.1)	23 (0.1)
Unknown	1,135 (5.8)	123 (2.3)	1,258 (5.1)
Ethnicity			
Not Hispanic	17,797 (91.3)	5,082 (94.3)	22,879 (91.9)
Hispanic	311 (1.6)	94 (1.7)	405 (1.6)
Unknown	1,395 (7.2)	213 (4.0)	1,608 (6.5)
Urine culture positive	4,360 (22.4)	1,171 (21.7)	5,531 (22.2)
CCI (median [IQR])	2.0 [0, 4.0]	1.0 [0, 3.0]	2.0 [0, 4.0]
Neutropenia^a^	503 (2.6)	138 (2.6)	641 (2.6)
Immunocompromised status	3,453 (17.7)	581 (10.8)	4,034 (16.2)

Notes. IQR, interquartile range; AAPI, Asian American/Pacific Islander; CCI, Charlson Comorbidity Index.

a Refers to absolute neutrophil count less than 1,500 within 2 days of urine test collection

**Figure 2.**
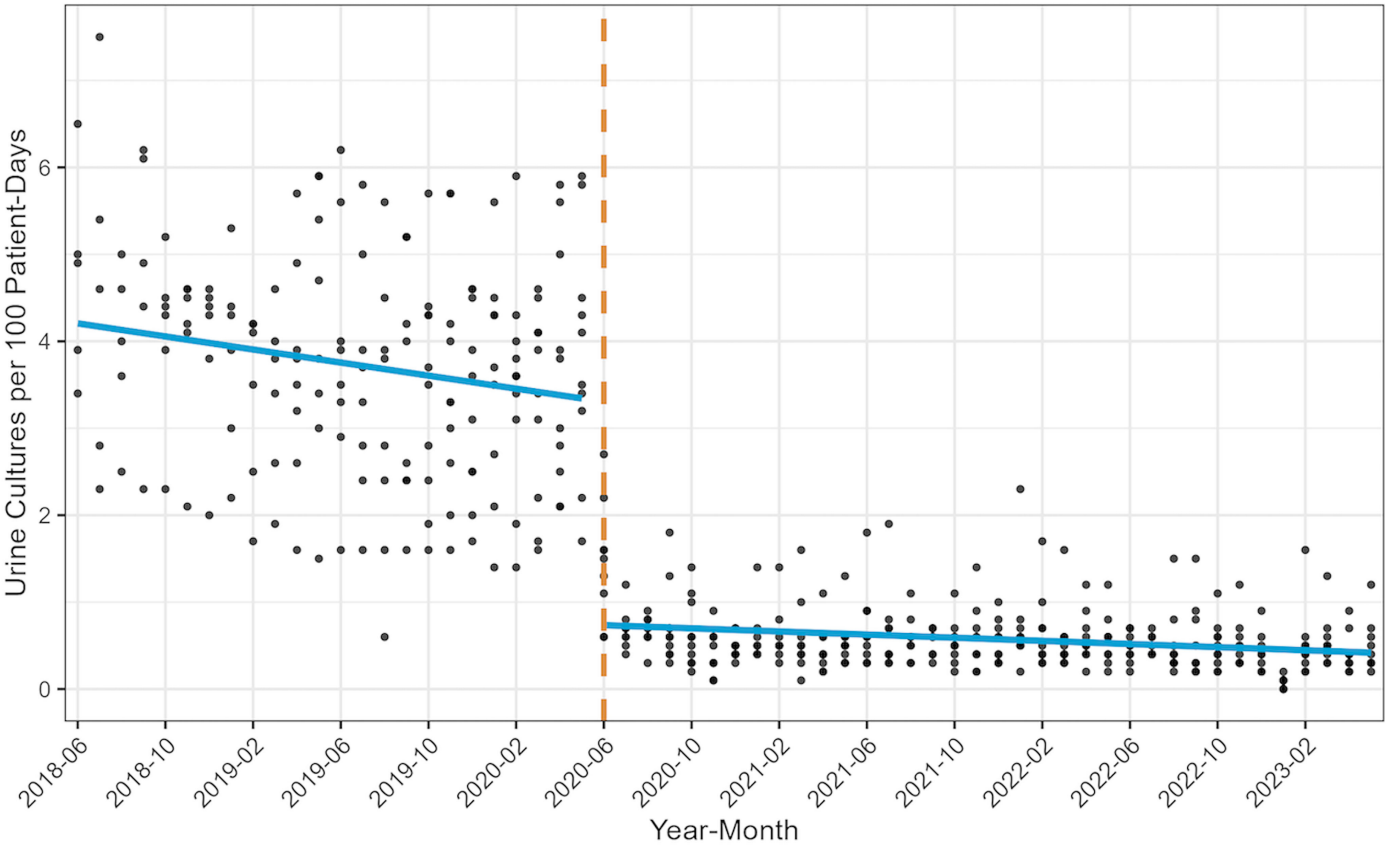
Mean urine culture rate pre- and post-intervention. Each point represents the monthly average number of urine cultures per 100 patient-days for each facility. Blue regression lines represent the line of best fit for all data points pre- and post-intervention. Dashed vertical line represents the intervention start date. (Study Part 1).

The mean rate of urine cultures per 100 patient-days by facility and month is shown in Figure 2. The mean urine culture rate per 100 patient-days for the entire pre-intervention and post-intervention periods system-wide was 3.71 and 0.54, respectively. The rate ratio of post- to pre-intervention urine culture rates was 0.143 or a decrease of 85.4% (95% confidence interval [CI] 0.139–0.148). A sensitivity analysis in which hospitals with any missing data were excluded (for a total of n = 15,576 cultures) yielded similar findings, with a rate ratio of 0.147 (95% CI 0.141–0.153)

Antimicrobial use data are summarized as follows: the total DOT within seven days of culture collection per 100 patient days was 39.18 in the pre-intervention group and 34.67 in the post-intervention group (rate ratio 0.885, 95% CI 0.865-0.904). The total DASC per DOT within the first seven days of culture was 11.37 versus 9.54 in the pre- versus post-intervention periods (rate ratio 0.839, 95% CI 0.833–0.845).

Among the 24,892 unique urine culture cases identified during the entire study, 19,361 had negative urine culture results, and of these, 4,240 occurred during the post-intervention period. Additionally during the post-intervention period, a cohort of 52,284 unique patient-cases with a negative UA (ordered separately or as UA which did not conditional reflex urine culturing) was identified to compare outcomes between patients excluded from urine culture based on normal UA and patients who ultimately were found to have a negative urine culture (ordered separately in a special population or as positive UA which conditionally reflex to urine culture). Outcomes were more favorable for all comparisons among negative UA cases among all clinical outcome categories (Table 2). When compared to post-intervention patients with negative urine culture, patients with negative UA in the post-intervention period had 82% lower odds of positive blood culture with a common urinary pathogen within 30 days of urine testing, 65% lower odds of mortality within 30 days of urine testing, a median hospital length of stay approximately 4 days shorter, and median hospital length of stay 0.3 days shorter when limited to patients with a length of stay of at least 48 hours. No meaningful difference in CDI rates was detected between pre-intervention (n = 496, 2.5%) and post-intervention (n = 116, 2.2%) groups (*P* = 0.11; not shown in table).

**Table 2.** Results of analysis of clinical outcomes during the post-intervention period, comparing negative urine culture to negative urinalysis without culture (Study Part 2)

Comparison	Negative urinalysis with no urine culture (n = 52,284)	Positive urinalysis with negative urine culture (n = 4,240)	Odds ratio (95% CI)	*P* value
Blood culture with urinary pathogen within 30 days^a ,^ ^b^	305 (0.58%)	134 (3.16%)	0.18 (0.15, 0.23)	<0.001
Mortality within 30 days^b^	1585 (3.03%)	367 (8.66%)	0.35 (0.31, 0.39)	<0.001
Median hospital LOS for all patients (days)	0.22^c^	5.25		<0.001
Median hospital LOS for patients with LOS ≥2 days (days)^d^	4.78^c^	5.44		<0.001

Notes. CI, confidence interval; LOS, length of stay.

a Pathogens could include *Escherichia coli* or any species of the genera *Klebsiella*, *Enterobacter*, *Proteus*, *Enterococcus*, or *Pseudomonas*.

b Beginning from date of urine test collection.

c Excludes 124 cases from urinalysis group without complete LOS data.

d Includes 14,771 urinalysis group cases and 4,096 urine culture group cases for a total 18,867 cases.

## Discussion

We demonstrate that a conditional reflex urine culturing intervention resulted in an 85.4% reduction in mean urine culture rate between pre- and post-intervention periods. We also observed an 11.5% decrease in DOT per patient-days and a 16.1% decrease in DASC/DOT within seven days of culture in the post-intervention group compared to the pre-intervention group, though this did not translate into a meaningful difference in CDI rates between pre-intervention and post-intervention groups (2.5 vs 2.2%, respectively). We did not observe higher rates of missed UTIs as a result of the intervention based on our comparison of post-intervention patients with negative urine cultures versus patients with negative UA not reflexed to culture obtained after the intervention, based on a lower observed rate of bacteremia with a urinary pathogen and 30-day mortality rate. Additionally, patients with UA not reflexed to culture post-intervention had a shorter hospital length of stay compared to those with a positive UA and negative urine culture. These findings suggest overall that the intervention decreased the frequency of urine cultures and antimicrobial usage without negative consequences among hospitalized adults. Interestingly, we did not observe a decrease in positivity rate following the implementation of conditional urine culturing, which may be reflective of the relatively conservative UA criteria required for culture.

Our findings are consistent with prior literature describing decreased urine culture frequency in the setting of urine culture reflex strategies. Howard-Anderson et al. and others describe urine culture reflex strategies using a UA with ≥10 white blood cells per high-power field (HPF) and found a 40–50% decrease in total urine culture orders per 1,000 patient days.^9,11,12^ Munigala et al. utilized a UA reflex to urine culture intervention based on positive nitrites or leukocyte esterase and demonstrated a 45% decrease in total urine culture order rate.^10^ Regarding antimicrobial utilization and urine culture reflex strategies, Watson et al. identified a significant reduction of 15% in DOT per 1,000 patient days while Sarg et al. noted only a 5% decrease.^11,13^ Fewer studies have evaluated potential negative consequences of urine culture reflex strategies. Penny et al. demonstrated only one case of sepsis occurring secondary to a missed UTI, while Caveness et al. showed there was no difference in pre- versus post-intervention rates of subsequent Gram-negative bacteremia.^17,18^

Our study adds to the growing body of literature describing laboratory interventions for urine culture and other antimicrobial stewardship optimization targets without adversely impacting clinical outcomes, such as specialized order sets or risk stratification for broad-spectrum coverage.^19,20^ Notable strengths of our study include large sample size and inclusion of multiple hospitals of varying size and patient population, and long pre- and post-intervention periods, increasing confidence in and generalizability of our findings. Additionally, we incorporated the DASC metric along with DOT, which confirmed there were no inadvertent effects of broader spectrum antimicrobial use occurring as a result of absence of microbiologic data.^16^ These findings are highly relevant to clinical practices, highlighting the intervention’s potential to reduce unnecessary antimicrobial use and strengthening the evidence for diagnostic stewardship practices as a powerful antimicrobial stewardship tool.

There are several limitations to acknowledge. First, we did not collect data on clinical manifestations in terms of documented signs of symptoms of UTI, though we did consider clinically relevant UTI outcomes including bloodstream infection with a urinary pathogen and mortality to establish the safety of this intervention. Due to the retrospective nature of our study and our reliance on matching data from multiple databases, some culture results could have been inadvertently lost during data extraction. However, any such losses would likely have occurred at random and similarly across pre- and post-intervention groups, making it unlikely that they introduced systematic bias or affected the overall validity of our findings which are based on a robust dataset. Another consideration for our study was that we did not analyze the potential impact on provider dissatisfaction caused by our intervention, which is an important outcome for the long-term effectiveness of automated and electronic medical record-based interventions. Further investigation into the broader impact of the laboratory intervention is warranted, including clinically relevant additional positive and negative outcomes over more extended periods such as adverse drug events and hospital readmissions, and in different subgroup populations such as those with more prolonged LOS.

## Conclusion

A conditional reflex urine culturing intervention led to a reduction in urine culture rate and DASC/DOT without observed negative consequences. Our results strengthen the evidence for reflex culture as an effective means of reducing urine culture rates and enhance our understanding of its safety and positive impact on antimicrobial use.
